# Youth and Adolescent Athlete Musculoskeletal Health: Dietary and Nutritional Strategies to Optimise Injury Prevention and Support Recovery

**DOI:** 10.3390/jfmk9040221

**Published:** 2024-11-05

**Authors:** Rebekah Alcock, Matthew Hislop, Helen Anna Vidgen, Ben Desbrow

**Affiliations:** 1 UQ College, The University of Queensland, Brisbane, QLD 4072, Australia; rebekah.alcock@uq.edu.au; 2 Total Fusion, Brisbane, QLD 4172, Australia; 3 Brisbane Sports and Exercise Medicine Specialists, Brisbane, QLD 4170, Australia; 4 School of Exercise and Nutrition Sciences, Faculty of Health, Queensland University of Technology (QUT), Brisbane, QLD 4072, Australia; h.vidgen@qut.edu.au; 5 Health Sciences and Social Work, Griffith University, Gold Coast, QLD 4222, Australia

**Keywords:** athletes, injury, rehabilitation, sports nutrition, dietary intervention, psychosocial considerations

## Abstract

**Background:** Despite the well-documented benefits of exercise and sports participation, young athletes are particularly vulnerable to musculoskeletal injuries. This is especially true during periods of rapid growth, sports specialisation, and high training loads. While injuries are an inevitable aspect of sports participation, the risk can be minimised by promoting the development of strong, resilient tissues through proper nutrition and injury prevention strategies. Moreover, targeted nutrition strategies can accelerate recovery and rehabilitation, allowing for a quicker return to sports participation. **Methods:** This narrative review synthesises scientific evidence with practical insights to offer comprehensive dietary recommendations aimed at strengthening tissues and supporting the healing process during recovery and rehabilitation. The selection of all sources cited and synthesised in this narrative review were agreed upon by contributing author consensus, experts in sports nutrition (R.A., H.V., B.D.) and exercise and sports medicine (M.H.). **Results:** Key topics include factors that contribute to injury susceptibility, general dietary recommendations for growth and development, sports nutrition guidelines, and nutrition considerations during injury and rehabilitation. This review also addresses external factors that may lead to suboptimal nutrition, such as food literacy and eating disorders. **Conclusions:** By highlighting these factors, this article aims to equip coaches, nutritionists, dietitians, athletic trainers, physical therapists, parents/guardians, sporting organisations, and schools with essential knowledge to implement effective nutritional strategies for injury prevention, recovery, and rehabilitation, ultimately enhancing long-term health and athletic performance.

## 1. Introduction

Youth and adolescent athletes are in a critical phase of both physical and psychological development. Regular exercise during this time provides numerous benefits, including social interaction, enhanced physical health and the development of self-identity and self-esteem [1]. The second decade of life is important for shaping an individual’s relationship with food and provides exposure to competitive sport, while individuals transition through dramatic biological, psychological, psychosocial, and academic/vocational changes [2]. For many aspiring athletes, the pathway to elite adult performance is typically uncertain, as success as a junior athlete rarely forecasts elite adult performance [3] and is likely to involve a combination of multifaceted elements [4]. Therefore, nutrition advice to developing athletes must prioritise long-term health.

Due to their rapid growth and development, youth and adolescent athletes are particularly susceptible to musculoskeletal injuries [5]. These injuries, whether acute or chronic, can have significant short- and long-term consequences on their health and performance. Several factors may increase the risk of injury in youth and adolescent athletes, including early sports specialisation; high volume training loads; and inadequate dietary intake to support growth, development, sports performance, and recovery [6,7,8]. These risks are further amplified by considerations such as poor training load management, a lack of education on proper technique, and inadequate strategies to mitigate the risk of overtraining.

Dietary factors, such as insufficient dietary intake to fully support growth and development, limited nutrition education (including sports-specific knowledge), low food literacy, or the presence of an eating disorder can increase the risk of injury due to inadequate nutrient intake [9]. Meanwhile, proper nutrition is essential during rehabilitation, ensuring that the necessary nutrients are available to support tissue repair [10].

This narrative review begins by examining key sports medicine considerations, including the prevalence and common types of injuries across different sports in youth and adolescent athletes. It will also highlight critical risk factors such as periods of rapid growth, improper training practices, early sports specialisation, and inadequate dietary intake. The review then transitions to discussing the specific nutritional requirements of youth and adolescent athletes that are necessary for developing strong, injury-resistant tissues. Additionally nutritional strategies for supporting injury management and rehabilitation will be addressed. The selection of all sources cited and synthesised in this narrative review were agreed upon by the consensus of contributing authors, experts in sports nutrition (R.A., H.V., B.D.) and exercise and sports medicine (M.H.).

Finally, the review explores the various factors that influence dietary intake within this group, including socio-economic, cultural, and psychological determinants, with a particular focus on the rising prevalence of eating disorders among young athletes. This comprehensive exploration incorporates elements of physiology, psychology, training science, and sociology. By providing a thorough overview of these factors, this review aims to serve as a foundation for practical and evidence-based interventions that can be utilised by coaches, nutritionists, dietitians, athletic trainers, physical therapists, parents/guardians, schools, and sporting organisations to support the long-term health and performance of youth athletes.

### 1.1. Prevalence of Sports Injuries

Determining exact prevalence rates of youth injuries is challenging due to several factors, including the increasing numbers of participants, the wide variety of sports, and the range of ages involved [11]. Most importantly, there is a lack of comprehensive databases to record injury occurrences [12]. Participation rates and documentation vary significantly by country and sport, further complicating evidence.

Nonetheless, documented rates of injuries in youth and adolescent athletes are still of significant concern. For example, in the United States, between 2015 and 2019, athletic trainers reported an injury rate of 2.29 injuries per 1000 athletic exposures [13]. In 2001, it was reported that sports-related injuries accounted for 2.6 million visits to US emergency rooms [14,15]. Data from the Australian Institute of Health and Welfare (AIHW) indicated that in 2021–2022, there were 18,700 injuries among children and adolescents from sport, exercise, or recreational activities [16]. A Canadian report indicated that 33–41% of youth have experienced at least one serious sport injury requiring medical attention, with data obtained during 2003, 2005, and 2007 [17].

Aside from reported injury rates, many more injuries are likely treated by a local allied health practitioner or general practitioner and may not present to emergency rooms, leading to under-reporting. As limited aggregation of this data is undertaken, our understanding of non-emergent (e.g., chronic) injuries is less comprehensive than those requiring acute care.

### 1.2. Common Types of Injuries

Young athletes are highly susceptible to a range of musculoskeletal injuries, with both the type and severity depending on the specific demands of their sport. Understanding these risks is crucial for developing effective prevention and treatment strategies that protect their still-developing musculoskeletal systems and prevent long term damage. High training loads and repetitive movements place significant stress on young athletes’ bodies, increasing the risk of both acute and overuse injuries.

For instance, repetitive throwing in baseball can lead to overuse injuries, like “thrower’s elbow” and osteochondritis dissecans, while fast bowlers can suffer from pars interarticularis stress fractures due to the high impact forces exerted on the lower back during bowling [18]. Similarly, sports involving jumping such as basketball, gymnastics, diving, and volleyball often result in Osgood–Schlatter disease, characterised by inflammation of the patellar tendon [19].

Growth plate injuries are another key concern, especially in weight-bearing and rotational sports [20]. For example, sustaining a slipped upper femoral epiphysis (hip) is common in these sports, while gymnasts often experience epiphyseal plate fractures (Salter–Harris fractures), due to high-impact forces, such as tumbling or during vaulting routines. Meanwhile, contact sports like rugby, American football, Australian rules football, and soccer are at increased risk of injuries such as concussions, as well as muscle and connective tissue tears and fractures [21].

In sports that involve significant running or jumping, such as track and field basketball, traction apophyseal injuries like Sever’s disease (heel) and Osgood–Schlatter’s disease (knee) are common [22]. Improper loading in sports also contributes to physeal trauma, such as Scheuermann’s disease in the thoracic spine, and stress fractures, particularly of the pars interarticularis, frequently occur in adolescents [23]. Additionally, sports that require sudden directional changes lead to common soft tissue injuries, including sprains, strains, and tendonitis [24].

### 1.3. Injury Risk Factors and Prevention Strategies

Youth and adolescent athletes are susceptible to both acute and long term (over-use) injuries, which can have substantial implications for their health and performance both in the short and long term. Several unique risk factors associated with growth, development, training practices, and nutrition contribute to this susceptibility. Implementing tailored preventative measures is essential for reducing injury risks and promoting long-term health.

#### 1.3.1. Growth and Development

During growth, different body segments develop at varying rates, which means the risk of injury can change as children and adolescents mature. Additionally, children of the same chronological age can significantly differ in their biological maturity, making some more prone to injury than others [8]. The musculoskeletal system of young athletes is also different from that of adults with injuries occurring at the weakest points, including growth plates and apophyseal attachments [9].

To mitigate injury risks during growth and development, it is essential to monitor these phases carefully and adjust training programs accordingly. Training regimes should be modified to prevent excessive strain on vulnerable areas, ensuring sufficient rest and recovery. Regular medical assessment is also critical, particularly when athletes are entering a training program. An initial medical assessment, followed by annual screening conducted by a doctor or sports physician, can help to identify early signs of injury, allowing for timely interventions to mitigate risk. Allied health professionals such as athletic trainers, physical therapists, podiatrists, and dietitians play a pivotal role in identifying potential issues and promoting good habits that may influence injury risk.

#### 1.3.2. Early Sports Specialisation and High Training Loads

Additional risk factors include single sport specialisation and increasing training loads, volume, and intensity, especially at an early age without adequate recovery. Giving special attention to development and training techniques, rather than emphasising competition and winning, may help to minimise or mitigate injuries [10]. While early sport specialisation may benefit highly technical sports like rhythmic gymnastics in achieving elite status, the intense training from age 12 in other sports like swimming, gymnastics, and baseball may increase the risk of overuse injuries and burnout [11]. Sports involving considerable biomechanical repetition can expose bones and joints to a single set of activities, leading to strain that exceeds normal growth and healing capabilities, thus increasing the risk of overuse injuries [12].

To prevent these injuries, managing training loads is critical. Coaches should monitor and plan training for growing athletes; adjusting time, intensity, duration, and training frequency; allowing for adequate recovery; and ideally rotating through body parts to avoid overloading certain joints. Additionally, investing time in teaching proper technique not only optimises sports performance but also reduces the risk of injury [25].

Female athletes, who tend to begin sports at a later age, are particularly vulnerable to certain injuries (e.g., anterior cruciate ligament (ACL) ruptures). These injuries are linked to the unique physical and developmental characteristics of female athletes, including differences in joint biomechanics and hormonal influences on connective tissues [23]. As such, targeted preventive strategies such as exercise-based programs that focus on strengthening key muscle groups and correcting faulty movement patterns are crucial in reducing female athlete injury risk.

#### 1.3.3. Organisational Rule Changes, Equipment Improvements, and Structured Programs

Injury surveillance studies have led to rule changes, equipment improvement, and strengthening regimens that help to prevent injury [20]. These initiatives are essential for reducing the risk and severity of injuries, promoting safer sports participation, and enhancing overall health and performance of young athletes [21]. For example, to reduce overuse injuries, baseball has adopted specific age-related limitations to the number and type of pitches allowed to be thrown. The USA baseball medical and safety advisory committee recommends limits on the number of pitches thrown per game and per week by young pitchers, along with mandatory rest periods to help prevent arm injuries [22].

#### 1.3.4. Inadequate Dietary Intake

Suboptimal nutrient intake during adolescence significantly impacts a young athlete’s ability to withstand physical stress, particularly during crucial periods of growth and development. Adolescence is marked by rapid growth where bones lengthen, lean body mass increases, and sexual maturation occurs [26]. Adequate intake of energy, protein, calcium, vitamin D, iron, and zinc are essential to support these physiological changes. Deficiencies in these nutrients can hinder growth and development, resulting in a weakened musculoskeletal system that is less resilient and more injury prone. For instance, insufficient calcium and vitamin D can impair bone mineralisation, increasing the risk of fractures [27]. Inadequate protein intake can also limit muscle repair and growth, reducing the bodies’ ability to withstand physical stress [28]. Iron deficiency, particularly common in adolescent females, reduces red blood cell production, leading to fatigue and decreased oxygen delivery to muscles, thus impairing recovery from physical exertion [29,30]. Furthermore, due to increased physical activity, which places additional stress on the musculoskeletal system, adolescent athletes may also require higher nutrient intakes than less active children and adolescents [9,31]. Therefore, meeting nutritional requirements for growth, development, and increased physical activity is crucial for building strong, resilient tissues that can withstand the demands of sports, while inadequacies heighten the risk of injury and long-term issues, such as reduced bone density and chronic musculoskeletal problems.

#### 1.3.5. Conclusion: A Multi-Faceted Approach to Injury Prevention

Injury prevention in youth and adolescent athletes requires a comprehensive approach that integrates proper training practices, load management, adequate nutrition, and recovery strategies. Monitoring growth and development, managing training loads, and implementing tailored exercise programs are crucial for reducing risks. Alongside this, proper nutrition plays an essential role in supporting tissue health and recovery. Collaboration between coaches; parents/guardians; and allied health professionals such as sports physicians, athletic trainers, physical therapists, and sports dietitians are essential for developing individualised injury prevention strategies. Combined with evidence-based nutrition strategies, these individuals can play a pivotal role in reducing the risk of injury in youth and adolescent athletes and promote long-term health and performance.

## 2. Nutrition to Support Injury Prevention

Nutrition plays a critical role in injury prevention by supporting the development of strong and resilient tissues, therefore helping to reduce the risk of both acute and overuse injuries. This section will provide an in-depth examination of the nutritional requirements specific to youth and adolescent athletes, outlining the general guidelines necessary to support growth, development, and athletic performance whilst minimising injury risk. Additionally, the concept of low energy availability (LEA) will be explored, as insufficient caloric intake relative to the demands of training can significantly impair tissue health and increase injury susceptibility [32].

### 2.1. General Nutritional Requirements for Growth and Development

#### 2.1.1. Human Growth Stages

Human growth is divided into three sequential components: infancy, childhood, and puberty (adolescence). Growth during puberty is directly related to hormonal changes that accompany sexual development and is characterised by three phases [33]:

1. Minimal height velocity (prepubertal growth lag).
2. Peak height velocity (PHV) (maximal growth).
3. Decreased height velocity (epiphyses fuse and final height is achieved).

Hence, adolescence is the period of maximal skeletal remodelling, with bones growing longer and subsequently hardening. Females start their growth spurt and attain PHV typically two years earlier than males (~12 years of age for girls vs. ~14 years of age for boys).

#### 2.1.2. Nutrient Demands and Utilisation

Nutrient demands and utilisation change considerably in response to pubertal growth. For example, increases in total bone mineral content can reach 1.1 g/day, while lean body mass gains can reach ~2.3 g/day in females and ~3.8 g/day in males, representing a threefold increase from the pre-pubertal period [34,35]. Providing dietary advice to support the deposition of key nutrients is important to optimise musculoskeletal health by early adulthood, which appears critical to reduce the subsequent risk of chronic conditions such as osteoporosis [35].

#### 2.1.3. Energy Requirements

Throughout adolescence, adequate energy is required to meet both the growth and development needs of the individual, as well as the substrate demands associated with general physical activity, training, and competition [36].

The energy expenditure of individual adolescent athletes can vary considerably due to changes in training and competition loads, participation in more than one competitive sport, part-time employment, and/or concurrent compensatory sedentary behaviours. The energy needs for growth consist of two parts: the energy expended to synthesise new tissues, and the energy deposited in growing tissues [36]. The energy expended to synthesize new tissues can be estimated via validated predictive resting metabolic rate (RMR) equations for developing athletes [37].

The energy deposited in growing tissues is considered small at ~2.0 kcal/gram of daily weight gain (e.g., for a 15-year-old male gaining 6 kg/year = ~33 kcal/day) [36]. Hence, while musculoskeletal growth may alter total caloric requirements, changes associated with physical activity and/or sports training are likely to have a much greater influence on total energy demands of adolescent athletes.

#### 2.1.4. Estimating Energy Requirements

Adolescent energy expenditure can be estimated by recording the type, intensity, and duration of exercise and, using the body weight of the individual, computing the energy cost using values of metabolic equivalents (METs) for specific activities [38]. Alternatively, wearable technologies incorporating accelerometers represent a relatively inexpensive alternative to estimate individual energy expenditure.

#### 2.1.5. Macronutrient Contributions

Meeting the energy needs of training and growth in developing athletes requires important contributions from all macronutrients. Current evidence suggests dietary protein intakes in excess of typical population recommendations are required to achieve net protein balance in adolescents and adults following high-intensity exercise. That is, the growth observed during adolescence is likely a consequence of greater anabolic sensitivity to and utilisation of dietary amino acids, and that no additional protein intakes (beyond those for adult athletes) are required [39].

In terms of carbohydrates (CHO), the duration and intensity of an exercise session determines CHO utilisation patterns and refuelling requirements, with these typically prescribed in g/kg body weight amounts [40]. Adolescent sports may involve unique features impacting CHO requirements. These included changes to game durations or race lengths, different competition formats (e.g., sports carnivals, representative competitions, trials), and concurrent participation in multiple sports.

Adequate fat-soluble vitamins and essential fatty acid intakes are important for hormone production and provide energy to support growth and maturation [41]. In particular, omega-3 polyunsaturated fatty acids appear critical for brain development and function [42]. In addition, evidence suggests that maximal fat oxidation rates (relative to lean mass) appear slightly higher in athletes <18 years [43].

#### 2.1.6. Micronutrient Requirements

Adolescent growth and development places emphasis on several micronutrients. Skeletal development relies on sufficient calcium with requirements directly associated with changes to skeletal growth [44]. Vitamin D status influences calcium regulation, in addition to impacting muscle and immune system function. Typically, fixed amounts of vitamin D are recommended beyond infancy, until values for older adults are further increased to account for the reduced capacity of the skin to produce vitamin D with ageing.

Similar to adults, young athletes are at high risk of vitamin D deficiency if they have little exposure to the sun—such as in those who live at latitudes >35 degrees, spend long periods training indoors, have dark skin, use sunscreen, or wear protective clothing [45]. Increases in red blood cell mass and menstrual losses elevate iron requirements during adolescence. Depleted iron stores (initial stage of iron deficiency) are observed frequently in studies on adolescent (particularly endurance) athletes [46]. Because growth increases iron requirements in adolescents compared to older athletes, the progression from low iron stores to a state of iron deficiency can be rapid.

Finally, zinc forms a component of various enzymes that help maintain structural integrity of proteins and regulate gene expression. Consequently, zinc deficiency during childhood has been shown to adversely affect brain growth, learning, and activity [47]. Dietary zinc requirements are associated with musculoskeletal growth.

### 2.2. Sports Nutrition Recommendations

Sports nutrition strategies for youth and adolescent athletes must support growth and development, while considering the specific demands of their sport. Adequate carbohydrate intake is vital for maintaining energy levels, particularly in high energy output sports. In contrast, strength-based sports require a focus on protein intake to support muscle repair and growth, especially as resistance training volumes typically increase with age [48]. However, research suggests that adolescents may have greater anabolic sensitivity compared to adults, meaning their protein needs may not be as high as those of their adult counterparts [39].

Despite the importance of sports-specific nutrition for youth and adolescent athletes, the research remains limited. This highlights the need for practitioners to fully understand both the physiological and training demands of each sport and the developmental processes in young athletes to create effective nutrition strategies that optimise performance and promote long-term health.

Table 1 provides an overview of key sports nutrition recommendations designed to support growth and development, while accounting for the nutrient demands associated with regular exercise. This section will also cover low energy availability (LEA) and relative energy deficiency in sport (REDs) in the context of youth athletes, emphasising their importance in injury prevention. Finally, the role of sports foods and supplements in young athletes will be discussed, providing guidance on their appropriate use.

#### 2.2.1. Low Energy Availability and Relative Energy Deficiency in Sport

Youth athletes have high energy demands, and if these are not met, there is a significant risk of developing LEA. Low energy availability occurs when there is insufficient energy to support the body’s physiological functioning after accounting for the energy expended during exercise [32]. This can lead to severe consequences, such as the development of relative energy deficiency in sport (REDs), a broader syndrome resulting from chronic LEA, affecting various physiological systems [52].

Although primarily studied in adults, LEA and REDs can significantly affect youth athletes. REDs can disrupt growth and development, leading to weak tissues, prone to injury. Chronic energy deficiency also weakens the immune system, making young athletes more susceptible to infections and illness. In females, LEA can disrupt normal hormonal balance, causing delayed menarche or amenorrhea, which affects bone metabolism and increases the risk of stress fractures, potentially progressing to osteopenia and osteoporosis [32,53]. Additionally, LEA is associated with mental health issues, including depression, anxiety, and irritability [54].

Diagnosing LEA and REDs in youth and adolescent athletes is challenging due to limited research and the absence of standardised diagnostic criteria [32]. Currently, diagnosis relies on observing clinical signs and symptoms, such as decreased training capacity (including reduced energy levels and muscle strength), unexplained and frequent injuries (including soft tissue, connective tissue, and bone stress injuries), vitamin or mineral deficiencies (e.g., iron and vitamin B12), menstrual irregularities, delayed menses, and mood disturbances [52]. A thorough dietary assessment conducted by trained clinicians, such as accredited sports dietitians, is essential for proper identification.

Common diagnostic tools, like testosterone levels and bone mineral density (BMD), may not be suitable indicators for youth and adolescent athletes due to significant variability of testosterone during puberty [55]. Additionally, the risk versus benefits of radiation exposure through dual-energy X-ray absorptiometry (DXA) for assessing BMD in children under 18 years of age should be considered, and routine scanning is not recommended [56].

Despite these challenges clinicians, parents/guardians, and coaches must be vigilant for symptoms of LEA and REDs in youth athletes. Monitoring changes in resting heart rate, unexplained fatigue, recurrent illness, mood disturbances, and dietary intake relative to energy expenditure can aid in early identification and intervention.

#### 2.2.2. Sports Foods and Supplements for Youth and Adolescent Athletes

Developing athletes have the potential for large performance gains through maturation and experience in their sport, along with adherence to proper training, nutrition, and rest regimens. As such, it is generally considered inappropriate for young athletes to be encouraged to consume dietary supplements [57,58]. This recommendation excludes the clinical use of dietary supplements for musculoskeletal repair/recovery (e.g., calcium, vitamin D, collagen) when administered under appropriate guidance from suitably qualified health professionals (e.g., a medical practitioner or dietitian).

Apart from issues related to safety (e.g., risk of contamination, mislabelling, unregulated ingredients, and potential adverse effects such as kidney or liver damage), the use of legal supplements in developing athletes over-emphasises their ability to manipulate performance and/or recovery [59]. Therefore, prioritising prudent training on the risks of supplementation, and the benefits of using a whole-food approach to meet nutritional requirements is essential.

## 3. Nutrition to Support Injury Management and Rehabilitation

When addressing the nutritional demands for youth and adolescent athletes post-injury, both the injury type and the stage of rehabilitation must be considered. It is not uncommon for athletes to reduce their intake post-injury due to decreased appetite, injury to the jaw and other body parts involved in eating, and the psychological impact of immobilisation and time away from sports. However, dietary restriction during this period should be cautioned against as it is crucial to optimise the body’s availability of specific nutrients to support tissue healing, regeneration, and ongoing growth and development [10,60]. This section will discuss general energy and macronutrient considerations, micronutrients, and collagen to support injury recovery in youth and adolescent athletes.

### 3.1. General Nutrition Considerations Post-Injury: Energy and Macronutrients

Post-injury, many athletes mistakenly reduce their energy intake due to reduced physical activity. However, resting energy needs increase during the early stages of injury because of heightened metabolic demands [61]. These increased demands persist despite lower levels of physical activity and vary depending on the severity of the injury, the complexity of surgery, and duration of immobilisation [36,60,61].

Trauma or surgery elevates RMR (i.e., energy expenditure at rest) by 20–50%. This increase is particularly pronounced during the initial hypermetabolic stage post-injury when the body is actively repairing tissue (e.g., after surgery or during bone remodelling) [62]. Energy requirements during this stage can be calculated using RMR, multiplied by activity and stress factors (e.g., RMR × activity factor × stress factor). After this initial phase, maintaining baseline energy requirements is recommended (e.g., RMR × activity factor) [61,62,63]. Limiting the intake of “empty calories” from processed, nutrient-poor foods is crucial to ensure that the body receives adequate nutrients to support ongoing healing, growth, and development [64].

Carbohydrates remain an essential energy source during injury recovery as they prevent the breakdown of lean mass and help to fuel tissue repair [10]. During recovery, carbohydrates spare protein from being used as an energy source, allowing essential amino acids (EAAs) to be used for muscle preservation and repair [65]. Emphasising low glycaemic index (GI), higher-fibre varieties such as wholegrains, fruits, vegetables, and legumes ensure sustained glucose and energy release; prevent hyperglycaemia; and maintain satiety, which can reduce the risk of overconsuming calories while ensuring adequate energy intake [62,66]. To ensure optimal intake without excessive energy consumption, carbohydrate intake should be in the lower range of standard sport nutrition recommendations (i.e., 3–5 g/kg BM) (see Table 1).

Protein is a critical macronutrient involved in the healing and regeneration of muscle tissue and may also have positive implications for bone health [28,67]. Furthermore, protein has been shown to play a role in preventing disuse atrophy by stimulating muscle protein synthesis (MPS) and reducing muscle protein breakdown (MPB) during periods of inactivity or immobilisation [68,69]. As rehabilitation progresses and activity increases, protein is vital for rebuilding lean mass and enhancing overall recovery [68]. Distributing protein intake throughout the day (e.g., 0.3 g/kg BM across five meals and snacks) provides the necessary EAAs to minimise muscle loss and support healing [39,61,68]. Supplemental sources are generally unnecessary for youth and adolescent athletes unless dietary intake is compromised. milk-based supplemental drinks can help to meet protein needs when dietary intake is inadequate. However, isolated protein supplements, such as whey protein isolate (WPI), are not recommended for youth and adolescent athletes because they can lead to excessive protein intake, displace the intake of other essential nutrients, and promote an over-reliance on supplements instead of whole foods. Ensuring adequate intake of lean meats, legumes, and dairy products will assist in meeting protein requirements.

Fats are essential for supporting recovery as they support bodily functions such as hormone production (e.g., steroid hormones like testosterone and estrogen) and the absorption of fat-soluble vitamins (i.e., A, D, E, and K) [70,71]. However, excessive intake of unhealthy fats, particularly those found in processed foods high in sugar and saturated and trans fats, should be discouraged. These fats have been shown to increase systemic inflammation, which can hinder the recovery process [72]. Fat intake should adhere to recommendations (see Table 1).

Although athletes often aim to reduce inflammation after injury, this response is crucial for healing. Inflammation signals the body to deliver immune cells, nutrients, and oxygen to the injured site, which facilitates tissue repair [73]. Therefore, aggressive strategies to suppress inflammation, such as high dose fish oil supplementation, are generally not recommended in the early stages of recovery as this may hinder the natural healing process [74]. Instead, a focus should be placed on the consumption of whole food sources of omega-3 such as salmon, flaxseed, chia seeds, walnuts, and fortified foods (i.e., eggs). Omega-3s from whole foods can modulate the body’s response to injury without excessively suppressing inflammation, which is essential for initiating tissue repair.

In certain cases, injuries or medications may reduce an athlete’s appetite or impair their ability to eat. For example, injuries to the jaw or mouth may limit food intake, or medication side effects such as nausea may limit intake. In these situations, offering nutrient-dense, easily digestible foods such as soft fruits, yoghurt, eggs, and soups can assist in ensuring adequate nutrient intake. Liquid nutrition options such as smoothies, milkshakes, and fortified drinks and soft foods may be necessary when solid food intake is difficult [75]. Small, frequent meals can help to maintain nutrient intake, without overwhelming an individual with a small appetite. Incorporating high-calorie foods such as nut butters, avocado, olive oil, and full-fat dairy products can provide more energy in smaller volumes [76]. If nausea is not a concern, enhancing the flavour and aroma of foods can also help to stimulate appetite, while bland foods are more palatable when nausea is present [77].

### 3.2. Micronutrients: Diet Quality vs. Supplementation for Youth Injury Recovery

Following injury, it is critical to consider specific micronutrient needs based on the type of injury, ensuring adequate intake for optimal healing while addressing any underlying biochemical or dietary deficiencies. For bone injuries, calcium and vitamin D are essential. Calcium is necessary for bone deposition, while vitamin D facilitates calcium absorption and regulates bone mineralisation, both of which are crucial for fracture healing. Additionally, magnesium plays a pivotal role in bone turnover, calcium homeostasis, parathyroid hormone regulation, and vitamin D activation [27].

For muscle injuries, micronutrients like iron and zinc are essential. Iron supports the delivery of oxygen to muscle tissue, supporting cellular energy production and muscle repair [29,30], while zinc is involved in protein synthesis and cell proliferation, essential for muscle regeneration [78]. Recently, vitamin D has also shown to be beneficial for muscle repair and regeneration after injury [79].

In the case of connective tissue injuries, vitamin C is crucial for collagen synthesis. It serves as a cofactor in the enzymatic reaction that cross-links collagen molecules, providing structural integrity to tendons and ligaments. A deficiency in vitamin C results in weakened connective tissue, leading to impaired healing in not only ligaments and tendons but also other tissues that contain collagen, such as skin and muscle [80]. Additionally inadequate vitamin C can increase the risk of infection due to its role in immune system function, which is particularly relevant for injuries that are at a higher risk of infection such as injuries with open wounds [81]. However, high-dose vitamin C supplementation is not recommended, as it may interfere with the oxidative stress response, potentially impairing the beneficial physiological adaptation during later stages of rehabilitation when training loads increase [82]. Adequate vitamin C intake can be easily achieved through the consumption of citrus fruits, strawberries, kiwifruit, and vegetables [83].

In general, micronutrient supplementation should only be considered when deficiencies are confirmed through blood tests, or if other factors are affecting intake. In general, diets rich in whole foods incorporating various food groups are sufficient to support tissue recovery. Adhering to dietary guidelines such as the Australian Guide to Healthy Eating (AGHE) [60] or the American dietary guidelines [84] can help to ensure that young athletes consume adequate nutrients to support injury recovery and long-term musculoskeletal health without relying on unnecessary supplementation [85]. If intake is compromised or a deficiency is confirmed, supplementation may be warranted to correct nutrient imbalances and optimise recovery.

### 3.3. Collagen for Injury Recovery in Youth Athletes: Exploring Food-Based Strategies for Tendon, Ligament, and Joint Health

Collagen, collagen-specific amino acids (e.g., proline, glycine, and lysine), and collagen peptides have shown promise in supporting tendon, ligament, and joint health. They promote collagen synthesis which helps to improve the strength and integrity of these tissues and have also been shown to reduce joint pain and improve overall joint function. However, whilst the research is promising for connective tissue health and joint function, research also suggests that it has limited effects on muscle tissue growth or repair [86,87]. Preliminary research suggests that consuming ~10–15 g of gelatine (a partially hydrolysed form of collagen) 30–60 min before rehabilitation exercises may support injury recovery when included as part of a structured rehabilitation program [88,89,90]. However, it is important to note that the current research has been conducted in cell cultures, animal models, and adults, so direct application to youth and adolescent athletes remains unclear, particularly regarding supplemental sources. Additionally, the safety and efficacy of collagen supplementation in children and adolescents have not yet been established. Given these uncertainties, a food-first approach may be more suitable. However, a challenge arises from the fact that the typical Western diet is low in collagen-rich foods. While bone broth is commonly cited as a natural collagen source, its amino acid content is highly variable, making it unreliable source for providing therapeutic dosages of collagen [91]. As a practical and more readily accessible alternative, using gelatine, combined with vitamin C-rich fruit juice to create gummies, could be served as a snack before rehabilitation exercises to potentially support tissue repair. This strategy offers a safer and more appropriate option for children and adolescents compared to direct supplementation.

## 4. Factors Influencing Dietary Intake

### 4.1. Understanding the Determinants on Food Choices in Youth Athletes

**Figure 1:**
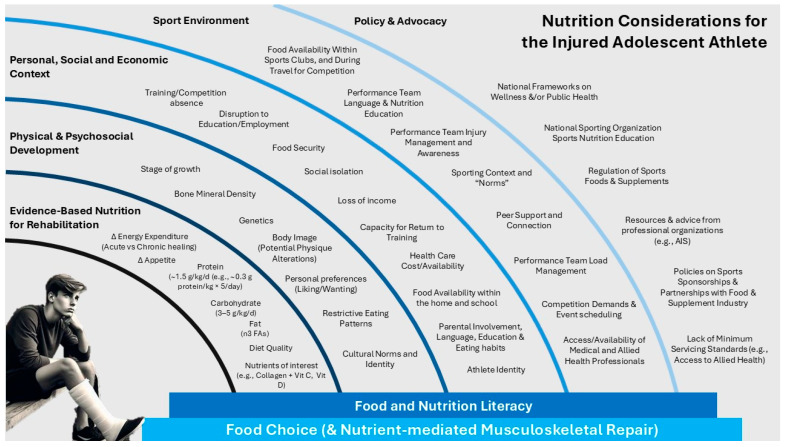
Nutrition considerations for the injured adolescent athlete. Image generated by Microsoft (2024) Copilot [AI software], https://www.microsoft.com/copilot, accessed on 1 July 2024.

Adhering to nutrition recommendations is challenging. Food choice is influenced by a variety of complex factors beyond individual willpower [92]. As illustrated in Figure 1, these influences operate at different levels.

At the macro level, food choice is influenced by food availability, with healthy food availability (especially fresh fruits and vegetables) being poor for many residents of lower socioeconomic class regions. Meso-level factors include the affordability of nutrition recommendations, personal socio-economic circumstances, and the resources available to families and communities. Micro-level individual influences such as balancing school; sports; social activities; taste preferences; food literacy; culture; social connections; identity; and the suggestions of more experienced teammates, parents/guardians, and coaches help determine daily food choices [2,93,94].

The process of food choice develops over a lifetime, creating values that help to interpret different situations and contexts [95]. In these varying contexts, adolescents will weigh up competing values like fitting in with peers versus eating for health, performance, or injury recovery when making food choices. Developing an adolescent’s food literacy can help increase their capacity to make informed choices [96]. Food literacy involves the knowledge, skills, and behaviours needed to plan, manage, select, prepare, and eat foods. Enhancing food literacy in adolescents makes following recommendations more likely by enabling adolescents to identify and select the right foods in the various environments. It also helps them to understand how to make these foods more palatable, social, and inclusive within their available resources (e.g., money, time, kitchen equipment) [97,98].

As there are many factors influencing food choices in young athletes, it is beyond the scope of this review to comprehensively address them all. That being said, medical conditions such as eating disorders pose a particularly serious concern impacting food choice in youth athletes.

### 4.2. Eating Disorders in Youth and Adolescent Athletes

Eating disorders present a serious risk to the health and development of young athletes. They compromise nutritional intake, hinder recovery, and impair musculoskeletal tissue health and development. Given the pressures youth athletes face, including social media influences, performance expectations, and athlete identity, it is crucial to recognise and address the risk of eating disorders for both health and injury prevention [99,100].

While eating disorders are often considered more prevalent among females, males also face significant risks, particularly when faced within unrealistic body ideals. Muscle dysmorphia is a subtype of body dysmorphic disorder characterised by an obsession with muscle size and a perceived lack of muscle mass, a subtype of body dysmorphic disorder characterised by an obsession with muscle size and a perceived lack of muscle mass [101,102]. Muscle dysmorphia is particularly common in sports that emphasise muscularity, like body building, which can lead to eating disorders and hazardous supplement use, such as anabolic steroids [103]. Additionally, sports emphasising weight and aesthetic appearance, such as gymnastics, combat sports, and athletics, are associated with a higher risk of developing disordered eating behaviours in both males and females [104].

An athlete’s identity (i.e., the degree to which an individual identifies with their role as an athlete [105]) can significantly impact their body image, often leading to heightened body dissatisfaction and dysmorphia. Pressures to meet physical ideals from coaches, parents/guardians, peers, and social media influencers can exacerbate body image concerns and promote unhealthy behaviours, potentially leading to clinically diagnosed eating disorders [106,107].

Although some disordered eating may occur in sports due to specific performance requirements (e.g., carbohydrate manipulation or low-fat diets), an eating disorder is considered clinical when it meets the criteria outlined in the Diagnostic and Statistical Manual of Mental Disorders, 5th edition (DSM-5). The DSM-5 categorises various eating disorders, often with overlapping symptoms. Common eating disorders including anorexia nervosa, bulimia nervosa, orthorexia, muscle dysmorphia, binge eating disorder, and avoidant/restrictive food intake disorder. For a full understanding on the diagnostic criteria, healthcare professionals should refer to the DSM-5 [108].

Athletes with an eating disorder may display a range of symptoms which vary depending on the disorders. Coaches, parents/guardians, and support staff should be aware of physical symptoms like rapid weight loss, weight fluctuations, fatigue, dizziness, gastrointestinal issues, decreased strength, reduced performance, an inability to maintain high intensities, frequent illness, and delayed recovery from injury. Behavioural signs may include changes in eating habits, excessive exercise, secretive eating, and a preoccupation with food [108].

The impact of an eating disorder on an athlete’s health is profound. Poor body image, leading to an eating disorder, often compromises nutritional intake, leading to deficiencies in energy (e.g., LEA), macronutrients, and micronutrients crucial for growth and development [109]. This increases the risk of fractures, strains, and sprains, both in the short and long term. Injury risk during training and competition also increases when LEA impairs concentration [52].

### 4.3. Practical Recommendations to Support Healthy Food Choices and Reduce the Risk of Eating Disorders in Youth Athletes

Practical recommendations for increasing food literacy in adolescent athletes include:

- Comprehensive sports nutrition education programs: Integrating comprehensive sports nutrition education into schools and/or sporting organisations, focusing on macronutrients, hydration, meal preparation, and meal timing. This education should also cover the risks and signs of eating disorders and highlight the importance of mental health alongside physical health and performance.
- Parent/guardian involvement: Encouraging parents/guardians to take an active role in fostering healthy eating habits by involving adolescents in meal planning, cooking, and grocery shopping. This helps build practical skills while reinforcing positive attitudes toward food and body image at home.
- Promoting realistic and healthy body standards: Fostering a culture of performance via health over appearance. Parents/guardians, coaches, and peers modelling body-positive attitudes that assist athletes to focus on achieving their personal best rather than conforming to unrealistic body ideals.
- Policy and advocacy: Advocating for policies that support access to healthy food options within schools and sports clubs, promoting the development of nutrition and mental health education programs to prevent disordered eating.
- Access to healthcare professional: Ensuring youth athletes have access to multidisciplinary teams including doctors, psychologists, and dietitians who can provide early intervention and ongoing support. This is especially important for athletes showing signs of eating disorders or those at risk of developing body image concerns.

## 5. Practical Applications

Based on the information provided throughout this review, the following section highlights key strategies and considerations to ensure the health, safety, and optimal growth and development of youth and adolescent athletes. Additionally, a visual representation illustrating key considerations for the injured athlete has been developed (Figure 1). That said, these suggestions serve as a guide only. Context-specific strategies should be guided by translational research frameworks (e.g., Knowledge to Action Framework (KTA) [110]) and/or behaviour change models (e.g., Capability, Opportunity, Motivation—Behaviour (COM-B) [111]) to support the implementation of evidence-based nutrition.

### 5.1. Prevention Strategies and Guidelines

This section outlines key factors influencing injury risk and strategies to mitigate these risks. It covers growth and development; understanding factors that increase injury risk; and addressing areas such as training optimisation, nutrition, and psychosocial influences. These are critical for creating a supportive environment that enhances athletic performance while minimising injury risk.

1. Background awareness
  - Understand the factors that can increase injury risk:
    - Growth spurts, inadequate nutrition, improper training loads, early sports specialisation, and psychosocial factors increase the risk of injury in youth athletes.
  - Understand the demands and common injuries associated with specific sports to develop tailored prevention strategies.
2. Individual factors
  - Monitor growth and development stages.
    - Educate athletes, coaches, and parents/guardians about different stages of growth and development and their implications for training.
    - Regularly assess physical development (tracking growth, height, weight, and skeletal maturity) and adjusting training programs to prevent injury.
  - Understand influences on food choices:
    - Recognise factors that influence food choices such as food availability, competing interests, taste preferences, sports nutrition and food literacy, culture, social connections, and athlete identity.
    - Tailor nutrition education to help athletes make informed food choices that meet their athletic performance and growth needs.
  - Optimise the environment.
    - Encourage policy changes in schools, sports clubs, and other environments to improve access to healthy, nutritious foods.
    - Promote the availability of nutritious meals and snacks that help support athletic performance and overall health.
  - Promote healthy body image and prevent eating disorders.
    - Foster a positive sports environment that emphasises health and performance over appearance.
    - Educate athletes on the risks of eating disorders and provide access to counselling and support services.
3. Training optimisation and health management
  - Monitor and manage training load.
    - Design training schedules that balance intensity, volume, and recovery periods appropriate for the athlete’s age and physical development.
    - Use methods to monitor training loads and make necessary adjustments to prevent overtraining and overuse injuries.
    - Progressively increase training intensity and volume with adequate rest periods to ensure safe progression and minimise injury risk.
  - Ensure proper technique and regular medical check-ups.
    - Ensure athletes receive adequate training on proper techniques.
    - Schedule regular medical and physical check-ups to monitor athlete health, identify potential risks, and provide modifications as necessary.
  - Prevent early sports specialisation.
    - Provide education on the risks of focusing on a single sport too early in an athlete’s career.
    - Encourage participation in various sports to support balanced athletic development.
4. Nutritional foundation
  - Establish a balanced diet which meets requirements for growth and development and prevent deficiencies.
    - Ensure athletes consume balanced amounts of energy, macronutrients, and micronutrients to meet demands of growth and development.
    - Provide education on food literacy skills such as shopping and cooking to support healthy eating habits.
  - Meet sports nutrition recommendations.
    - Assess energy needs based on the athlete’s growth stage and activity levels to prevent LEA and REDs.
    - Ensure adequate intake of macronutrients (carbohydrate, protein, and fats) to support fuelling, recovery, and repair.
    - Regularly check for and address any deficiencies considering the increased nutrition demands of youth athletes.
    - Educate athletes, coaches, and their parents/guardians on the importance of maintaining a diet that meets the nutritional needs of a growing athlete.

### 5.2. Management Strategies and Guidelines

This section focuses on the management of injuries in youth athletes, emphasising a multi-disciplinary approach. It provides strategies to support injury management, rehabilitation, and nutritional support to aid in recovery.

1. Injury management and rehabilitation
  - Develop injury care protocols.
    - Establish clear protocols for initial injury assessment and care.
    - Design structured rehabilitation programs tailored to the specific needs of the athlete and the injury, with nutritional support.
  - Utilise a multi-disciplinary approach for effective rehabilitation.
    - Involve a team of healthcare professionals, including athletic trainers, physical therapists, school nurses, dietitians, and mental health experts, in the injury management and rehabilitation process.
    - Regularly review and adjust rehabilitation plans based on progress and feedback from the healthcare team.
2. Nutritional strategies for injury and rehabilitation in youth and adolescent athletes
  - Tailor nutrient intake to support the injury type and stage of rehabilitation.
    - During recovery, energy needs initially increase to support tissue repair; therefore, preventing severe dietary restriction is essential.
    - Adequate macronutrient intake helps to ensure adequate intake of energy for healing, supporting hormone balance, preventing excessive inflammation, and reducing disuse atrophy.
    - Monitor for and assess nutrient deficiencies, particularly those that are specific to the injury type to ensure that the healing process if supported
    - Emphasise the importance of nutrient-dense, whole foods over supplements to support recovery and health. Supplements should only be used under the guidance of a healthcare professional.

## Figures and Tables

**Table 1:** Dietary recommendations for adolescent athletes undertaking training/competition.

Dietary Variable	Recommendation	Comments/Considerations
Energy	Total energy expenditure = RMR * + PA * RMR (kcal/day) = 11.1 × body mass (kg) + 8.4 × Height (cm)—(340 male or 537 female) (Reale et al., 2020) [37].	PA estimated via activity log (Ainsworth 2014) [38] or wearables (no ideal device). Devices tend to underestimate energy expended in some activities—incline walking, cycling, and carrying items. More accurate when placed close to the centre of mass (e.g., hip vs. wrist or ankle). ^†^
Protein	~1.5 g/kg/d (e.g., ~0.3 g protein/kg × 5 mealtimes)	Mazzulla et al., 2018 [39].
Carbohydrate	Training Light 3–5 g/kg BM Moderate (1 h/day) 5–7 g/kg BM High (1–3 h/day) 6–10 g/kg BM Acute CHO optimisation Prior to exercise: 1–2 g/kg 1–4 h before During exercise: 0 g/h in events ≤45 min 30 g/h in events 45–60 min 60 g/h in events 60–120 min Immediately after exercise: 1 g/kg/h for 2–4 h	Adapted from Burke L.M., et al. (2011) [49]. Most events undertaken by adolescent athletes are <120 min in duration.
Fat	20–35% of total energy Saturated and trans fats < 10%	Currently no specific recommendations for fat intake for athletes. Population reference standards used (NHMRC 2006) [44].
Calcium	9–11 years 1000 mg/day 12–18 years 1300 mg/day	Currently no specific recommendations for calcium intake for athletes. Population reference standards used. Absorption may be impaired by phytates, oxalates, and high caffeine doses. UK, 12–18 years = 800 mg/day.
Vitamin D	Australia 5 µg/day Brazil/USA 15 µg/day Canada 10–25 µg/day Europe 10–20 µg/day	Determined geographically and likely to be influenced by several lifestyle factors.
Iron	9–13-year-olds, 8 mg/day 14–18-year-old boys, 11 mg/day 14–18-year-old girls, 15 mg/day	US, 9–13-year-olds, 10 mg/day. Specific cut-offs for iron deficiency do not exist for adolescents. Individuals consuming non-haem iron sources (e.g., vegetarians) may require higher intakes due to lower bioavailability.
Zinc	9–13-year-olds, 8 mg/day 14–18-year-old boys, 11 mg/day 14–18-year-old girls, 9 mg/day	EU, 15–17-year-old boys, 14 mg/day. 15–17-year-old girls, 12 mg/day.

BM = body mass, PA = physical activity. ^†^ Conclusion from authors of reviews summarising the validity of different devices in younger populations [50,51].

## Data Availability

Not applicable.
